# Development of an Interaction Assay between Single-Stranded Nucleic Acids Trapped with Silica Particles and Fluorescent Compounds

**DOI:** 10.3390/jfb3030601

**Published:** 2012-09-05

**Authors:** T. Isoda, R. Maeda

**Affiliations:** 1 Department of Life and Environment Engineering, Faculty of Environmental Engineering, University of Kitakyushu, 1-1, Hibikino, Wakamatsu, Kitakyushu 808-0135, Japan; 2 Department of Life and Environment Engineering, Graduate School of Environmental Engineering, University of Kitakyushu, 1-1, Hibikino, Wakamatsu, Kitakyushu 808-0135, Japan; Email: s1mab008@eng.kitakyu-u.ac.jp

**Keywords:** nucleic acid, polynucleotide, assay, fluorescence, cellstain-AO

## Abstract

Biopolymers are easily denatured by heating, a change in pH or chemical substances when they are immobilized on a substrate. To prevent denaturation of biopolymers, we developed a method to trap a polynucleotide on a substrate by hydrogen bonding using silica particles with surfaces modified by aminoalkyl chains ([A-AM silane]/SiO_2_). [A-AM silane]/SiO_2_ was synthesized by silane coupling reaction of N-2-(aminoethyl)-3-aminopropyltrimethoxysilane (A-AM silane) with SiO_2_ particles with a diameter of 5 μm at 100 °C for 20 min. The surface chemical structure of [A-AM silane]/SiO_2_ was characterized by Fourier transform infrared spectroscopy and molecular orbital calculations. The surface of the silica particles was modified with A-AM silane and primary amine groups were formed. [A-AM silane]/SiO_2_ was trapped with single-stranded nucleic acids [(Poly-X; X = A (adenine), G (guanine) and C (cytosine)] in PBS solution at 37 °C for 1 h. The single-stranded nucleic acids were trapped on the surface of the [A-AM silane]/SiO_2_ by hydrogen bonding to form conjugated materials. The resulting complexes were further conjugated by derivatives of acridine orange (AO) as fluorescent labels under the same conditions to form [AO:Poly-X:A-AM silane]/SiO_2_ complexes. Changes in the fluorescence intensity of these complexes originating from interactions between the single-stranded nucleic acid and aromatic compounds were also evaluated. The change in intensity displayed the order [AO: Poly-G: A-AM silane]/SiO_2_ > [AO:Poly-A:A-AM silane]/SiO_2_ >> [AO:Poly-C:A-AM silane]/SiO_2_. This suggests that the single-stranded nucleic acids conjugated with aminoalkyl chains on the surfaces of SiO_2_ particles and the change in fluorescence intensity reflected the molecular interaction between AO and the nucleic-acid base in a polynucleotide.

## 1. Introduction

Recently, protein-function arrays and/or functional proteomics have attracted attention as a novel biotechnology that can obtain information about the interaction between a protein and another protein, a nucleotide or a small molecule. To construct this assay, it is necessary to immobilize many kinds of protein on a substrate. Conjugation of proteins and biopolymers with a substrate is a breakthrough technology for the development of protein-function arrays. Many studies of surface chemistry related to biomolecules have been reported. 

Physical adsorption has been widely used as an immobilization method [1,2]. When a solution containing protein is added dropwise onto a nitrocellulose [1] or polyvinylidene fluoride membrane [2], the protein is immobilized on the membrane surface by hydrophobic interactions; however, the adhesion is weak. Schreibe *et al*. chemically bonded bovine serum albumin (BSA) on a glass substrate using aldehyde and then further immobilized other proteins on this BSA-modified surface [3]. The carboxyl groups of the glutamate residues in BSA were converted to an active ester, which bonded to amino groups in other proteins. A different method was reported by Zhu *et al*. [4] who immobilized proteins on a glass substrate modified with epoxy groups through chemical bonding to amino groups in the proteins.

Jung and coworkers immobilized peptides on the surface of a gold electrode coated with a self-assembled membrane (SAM) [5]. In this case, the end of the SAM, which consisted of p-hydroxyazobenzene groups, was changed to *p*-quinonimine by electrolysis and oxidation. Cysteine residues in the peptide reacted with *p*-quinonimine by 1,4-Michael addition. Meanwhile, Byeon *et al*. immobilized antibodies on a substrate through condensation of hydrazine and aldehyde in the presence of aniline as a catalyst [6].

We have developed polystyrene (PS) microbeads, modified with schizophyllan (SPG) or polysaccharide [7]. The molecular weight of SPG was about 150,000 and its end unit was modified with an amino group. The amino group of SPG bound covalently to a carboxyl group on the support surface to form the composite PS∙SPG [8]. Nucleic acids were preferentially adsorbed in particular, and could be detected by the microelectrode when the adsorbent consisted of PS microbeads with a SPG-modified surface. 

A different protein immobilization method involves using a protein implanted with a ligand that acts as a tag [9]. The tag consisted of a continuous sequence of seven to eight histidine residues and could bond to a Ni complex on the surface of a substrate. An advantage of this method is that all proteins coordinate in the same direction, which prevents deactivation. It has been reported that 80% of 5800 kinds of yeast proteins maintained their activity when immobilized using this method. Katayama *et al*. also reported immobilization of an estrogen-receptor [10] and transcription factor [11] on a gold electrode using this method.

However, basic research investigating conjugation of a protein or biopolymer to a solid surface is not always suitable for application to protein-function array technology, although various methods have been developed. In particular, most immobilization methods use covalent bonding and/or hydrophilic interactions between a protein or biopolymer and a solid surface. There are few examples of immobilization methods using hydrogen bonding.

In this study, we fabricated silica particles with surfaces that were modified by aminoalkyl chains. The amino groups introduced on the surface can interact with single-stranded nucleic acids through hydrogen bonding, resulting in a conjugate between a polynucleotide and inorganic material. The single-stranded nucleic acid trapped to the silica particle was further interacted by an acridine orange derivative as a fluorescent label, allowing changes in fluorescence intensity originating from interactions between the single-stranded nucleic acid and aromatic compounds to be investigated. The affinity of an acridine orange derivative to interact with single-stranded nucleic acid trapped on the silica particle is discussed.

## 2. Experimental Procedures

### 2.1. Preparation of SiO_2_ Particles Modified with Aminoalkyl Chains

**Figure 1:**
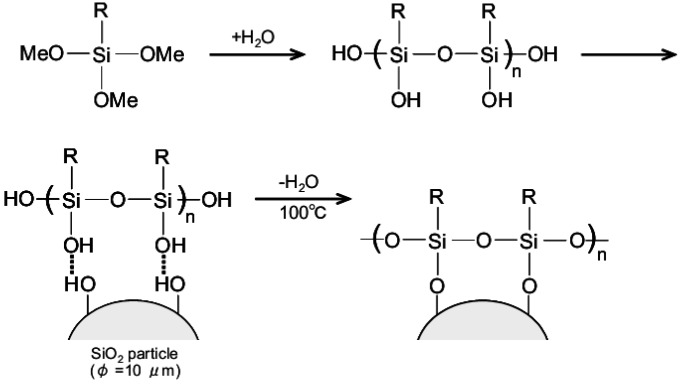
Reaction mechanism of silane coupling on a SiO_2_ particle.

**Figure 2:**
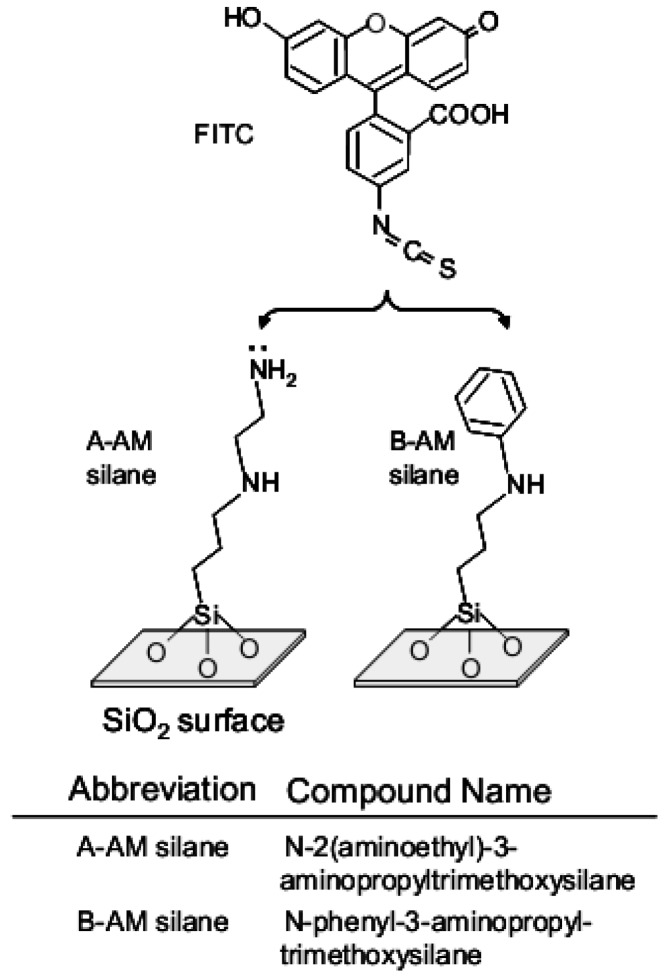
Reaction scheme of fluorescence labeling after preparation of two kinds of silane coupling on SiO_2_ particles.

Reaction mechanism of silane coupling on a SiO_2_ surface is shown in Figure 1 [12]. Hypersil silica (GL Science Co., Ltd., Japan; particle diameter: 5 μm) was used as SiO_2_ particles. Two kinds of silane derivatives were used; N-2-aminoethyl-3-aminopropyltrimethoxysilane (KBM-603; abbreviated to A-AM silane) and N-phenyl-3-aminopropyltrimethoxysilane (KBM-573; abbreviated to B-AM silane). Both chemicals were purchased from Shin-Etsu Chemical Co., Ltd., Japan. The structure was shown in Figure 2. Silanol derivatives were formed by hydrolysis of silane derivatives in aqueous solutions containing 2%, 20% and 40% A-AM or B-AM silane at 20 °C for 30 min. The silane coupling reaction of each silanol derivative was carried out at 100 °C for 10 min in a Teflon beaker. The hydroxyl groups of silanol derivatives were covalently bonded to hydroxyl groups on the SiO_2_ surface via condensation reaction to form SiO_2_ particles modified with aminoalkyl chains, [X-AM silane]/SiO_2_ (X = A: amino group and B: phenyl group).

### 2.2. Analysis of Surface Chemical Structure

Functional groups on the surface of the aminoalkylated SiO_2_ particles were qualitatively analyzed by Fourier transform infrared spectroscopy (FT-IR; Perkin Elmer, USA). Spectra were recorded using KBr discs containing 1 to 5 wt% sample. The substitution of aminoalkyl chains on the SiO_2_ surface was further confirmed by fluorescence labeling. The reaction scheme is also illustrated in Figure 2. Each sample (5 mg) and fluorescein isothiocyanate (FITC; 2.5 μg/mL, Dojindo Laboratories Co., Ltd., Japan) in PBS solution was stirred at 20 °C for 5 min. The sample was washed twice with distilled water (DW) to give labeled aminoalkylated SiO_2_ particles [FITC:X-AM silane]/SiO_2_ (X = A: amino group and B: phenyl group). Fluorescence intensity was determined using a fluorescence microscope (U-RFLT50, Olympus, Japan) at a wavelength of 520 nm. [FITC:X-AM silane]/SiO_2_ (5 mg) was dispersed in DW (300 μL) and placed in one well of a culture slide (well size: 10 mm^2^, BD Co., Ltd., USA). After deposition of the particle solution for 5 min, images were obtained at 15 arbitrary points in the cell and analyzed to determine average intensity.

### 2.3. Trapping Procedure of Single-Stranded Nucleic Acids Using Aminoalkylated SiO_2_ Particles

**Figure 3:**
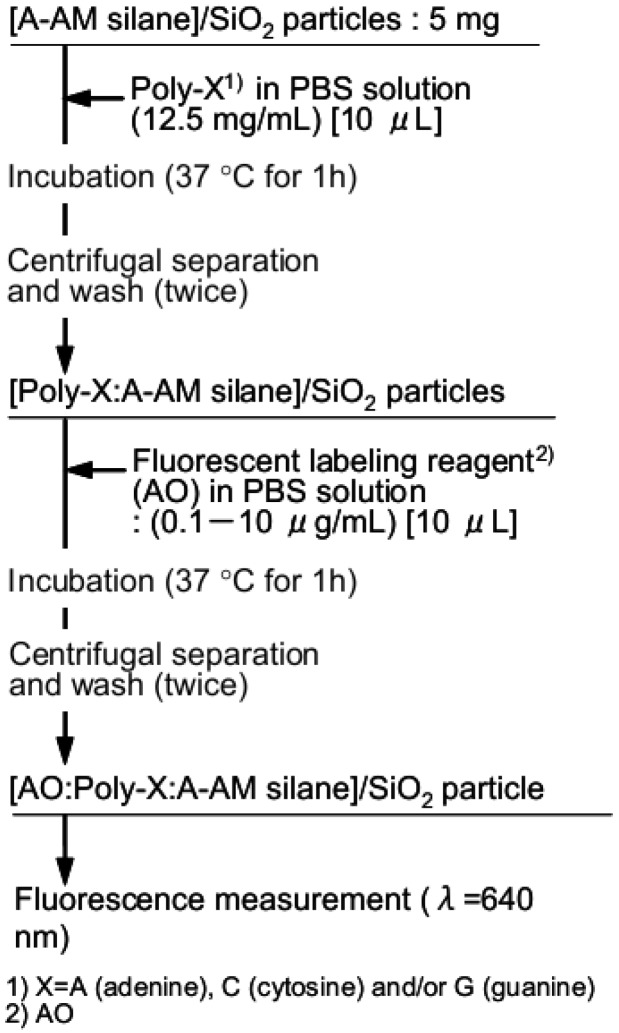
Flow chart outlining the preparation of [AO:Poly-X:A-AM silane]/SiO_2 _complexes for fluorescence analysis.

**Figure 4:**
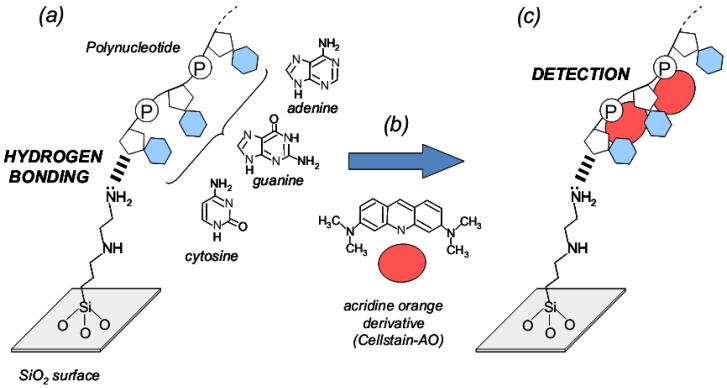
The schematic illustration of interaction assay between single-stranded nucleic acids trapped with silica particles and fluorescent compounds. (**a**) Stage of nucleotide trapping on aminoalkylated SiO_2_ particles; (**b**) structure of cellstain-AO as fluorescent compound; (**c**) and conjugation stage of cellstain-AO with single-stranded nucleic acids.

A flow chart outlining the preparation of complexes of single-stranded nucleic acid trapped with [A-AM silane]/SiO_2_ and fluorescence compounds is shown in Figure 3. The schematic illustration was also shown in Figure 4. Single-stranded nucleic acids composed of only cytosine, adenine or guanine (poly-C, poly-A and poly-G, respectively; Wako Chemicals, Japan) were used. Solutions of poly-X (X = C, A or G, 12.5 mg/mL) in PBS were prepared. A mixture of each poly-X solution and [A-AM silane]/SiO_2_ (5 mg) was incubated at 37 °C for 1 h. The particles were separated by centrifugation and then washed to give complexes of polynucleotide and [A-AM silane]/SiO_2_ ([Poly-X:A-AM silane]/SiO_2_; Figure 4a). An acridine orange derivative (named AO) was used as a fluorescent label (Cellstain-AO; Dojindo Laboratories Co., Ltd., Japan). The chemical structure is shown in Figure 4b. When AO interacts with phosphate group and/or nucleobase in single-stranded polynucleotides, such as complementary DNA or messenger RNA, the molecules are arranged in a random order. Some associate with AO in the single-stranded polynucleotide, giving rise to red fluorescence (λ_max_ = 650 nm) [13,14]. [Poly-X:A-AM silane]/SiO_2_ (5 mg) was dispersed in AO in PBS solution (0.1–10 μg/mL) and incubated at 37 °C for 1 h. The particles were separated by centrifugation and washed to give fluorescent-labeled [Poly-X:A-AM silane]/SiO_2_ particles ([AO:Poly-X:A-AM silane]/SiO_2_; Figure 4c). Fluorescence intensity was measured using a fluorescence microscope at a wavelength of 640 nm using the method described in the previous section.

### 2.4. Molecular Orbital Calculations of Surface Model Structure of Aminoalkylated SiO_2_, Polynucleotides and Fluorescent Compound

Molecular orbital (MO) calculations to obtain a surface model structure of aminoalkylated SiO_2_, polynucleotides and fluorescent compound were carried out using commercial software (MOPAC Vr.3, Fujitsu Co., Japan) with the PM3 method [15]. A-AM silanol as a coupling precursor and a Si_4_O_4_(OH)_10_ cluster combined with A-AM silanol as a surface model of a SiO_2_ particle is shown in Figure 6a and b, respectively (silicon and oxygen atoms are shown as yellow and red color). 

The geometry of each molecule was optimized, and then a hydroxyl group in each molecule was bonded to silica. The geometry was optimized again to generate an [A-AM silane]/SiO_2_ cluster. Simulated FT-IR spectra were determined by vibration calculation of the [A-AM silane]/SiO_2_ cluster using the PM3 method. Polynucleotide composed of three units of nucleobase and cellstain-AO molecule as a fluorescent compound was also calculated by the method described above.

## 3. Results

### 3.1. Chemical Structure of Aminoalkylated SiO_2_ Surface

**Figure 5:**
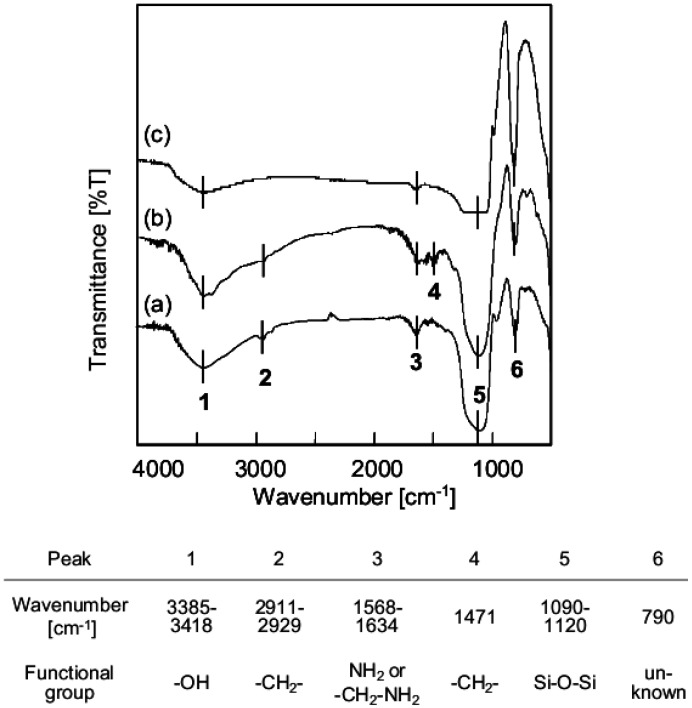
FT-IR spectra of the surface of SiO_2_ particles following amino-silane coupling. SiO_2_ particles treated with X-AM silane (n%) are abbreviated as [X-AM silane (n%)]/SiO_2_). (**a**) [A-AM silane (2%)]/SiO_2_; (**b**) [A-AM silane (40%)]/SiO_2_; (**c**) [B-AM silane (2%)]/SiO_2_.

FT-IR spectra of the surface of SiO_2_ particles after aminosilane coupling are presented in Figure 5. Here, SiO_2_ particles treated with X-AM silane (n%) are abbreviated as [X-AM silane (n%)]/SiO_2_. Figure 5a shows an FT-IR spectrum of [A-AM silane (2%)]/SiO_2_. The peaks labeled 1 and 5 are assigned to -OH (ν = 3385–3418 cm^−1^) and Si-O-Si bonds (ν = 1090–1120 cm^−1^) in SiO_2_. Peaks 2 and 3 are assigned to -CH_2_- (ν = 2911–2929 cm^−1^) and -NH_2_ or -CH_2_-NH_2_ bonds (ν = 1568–1634 cm^−1^), respectively. Figure 5b shows an FT-IR spectrum of [A-AM silane (40%)]/SiO_2_, which contains Peak 4 assigned to -CH_2_- bonds (ν = 1471 cm^−1^). These results suggest that A-AM silane has been introduced onto the surface of SiO_2_. Figure 5c shows an FT-IR spectrum of [B-AM silane (2%)]/SiO_2_ for comparison. In this case, peak 3 almost disappeared because there is no amino group in B-AM silane.

**Figure 6:**
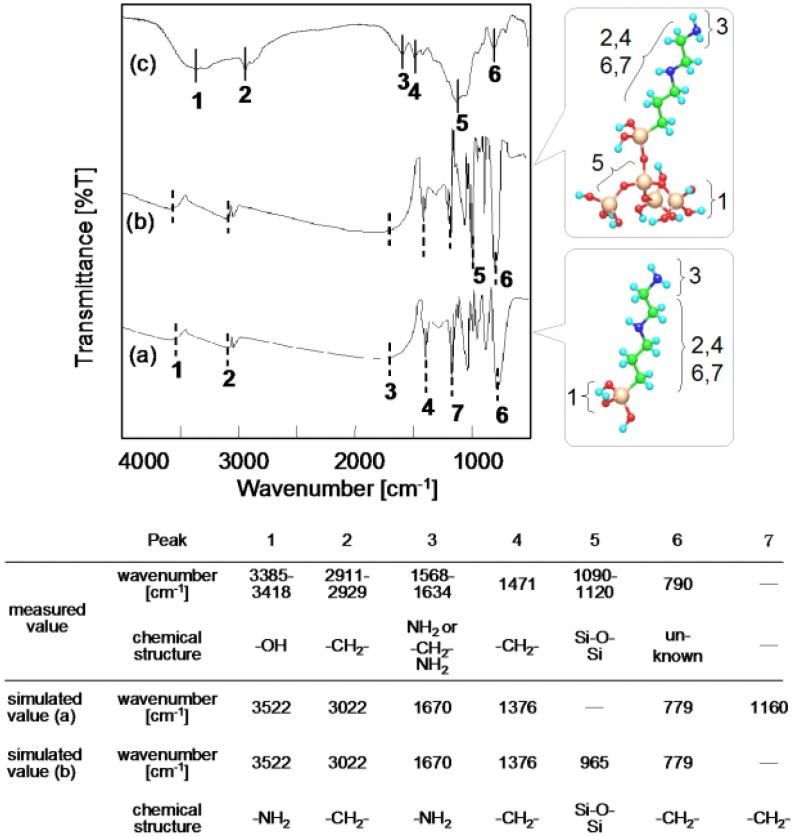
Simulated FT-IR spectra of (**a**) A-A silanol; and (**b**) Si_4_O_4_(OH)_10_ cluster combined with A-AM silane; (refer to Figure 2 for structures) compared to (**c**) measured FT-IR spectrum of [A-AM silane (40%)]/SiO_2_.

Measured and calculated FT-IR spectra are presented in Figure 6, where a spectrum measured for [A-AM silane (40%)]/SiO_2_ (Figure 6c) is compared to simulated spectra for A-AM silanol (Figure 6a) and [A-AM silane]/SiO_2_ clusters (Figure 6b). 

Six major peaks were obtained from the vibrational calculation for A-AM silanol, as shown in Figure 6a. Peaks 2, 4, 6 and 7 are assigned to -CH_2_- bonds (ν = 3022, 1376, 1160 and 779 cm^−1^), while peaks 1 and 3 are assigned to -NH_2_ bonds (ν = 3,522 and 1,670 cm^−1^). Except for peak 5, these correspond to the calculated spectrum for an [A-AM silane]/SiO_2_ cluster (Figure 6b). Peak 5 for the cluster is consistent with vibration of Si-O-Si bonds (ν = 965 cm^−1^). The wavenumbers of the simulated peaks are close to those in an FT-IR spectrum measured for [A-AM silane (40%)]/SiO_2_ (Figure 6c). 

**Figure 7:**
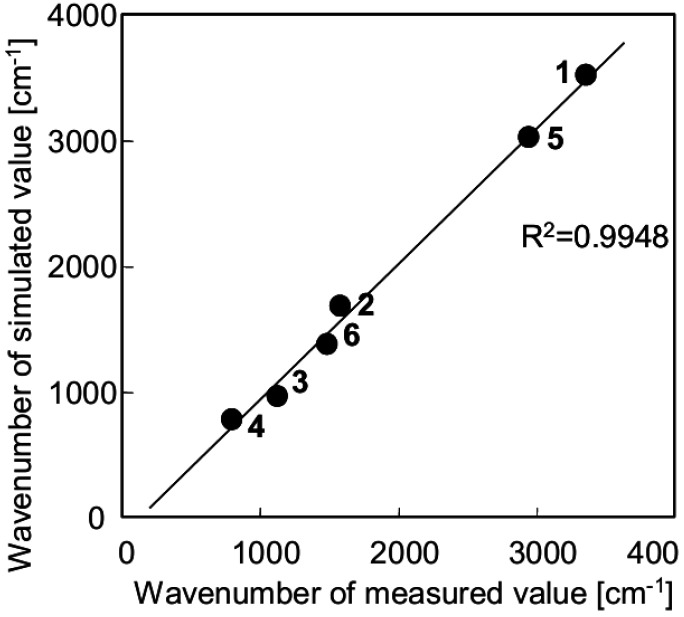
Relationship between the vibrational calculation result for a Si_4_O_4_(OH)_10_ cluster combined with A-AM silane (structure given in Figure 6b) and measured FT-IR spectrum of [A-AM silane (40%)]/SiO_2_.

Figure 7 shows the relationship between the results of the vibrational calculation for [A-AM silane]/SiO_2_ cluster as a model molecule (Figure 6b) and the measured data for [A-AM silane (40%)]/SiO_2_ (Figure 6c). There is a good correlation between the simulated and measured peaks. Fitting the data with a linear approximation gave a correlation coefficient of 0.9948. This suggests that the structure of the aminoalkyl chains on the SiO_2_ surface is similar to the structure predicted for the [A-AM silane]/SiO_2_ cluster. 

### 3.2. Orientation of Aminoalkyl Chains on the SiO_2_ Surface

**Figure 8:**
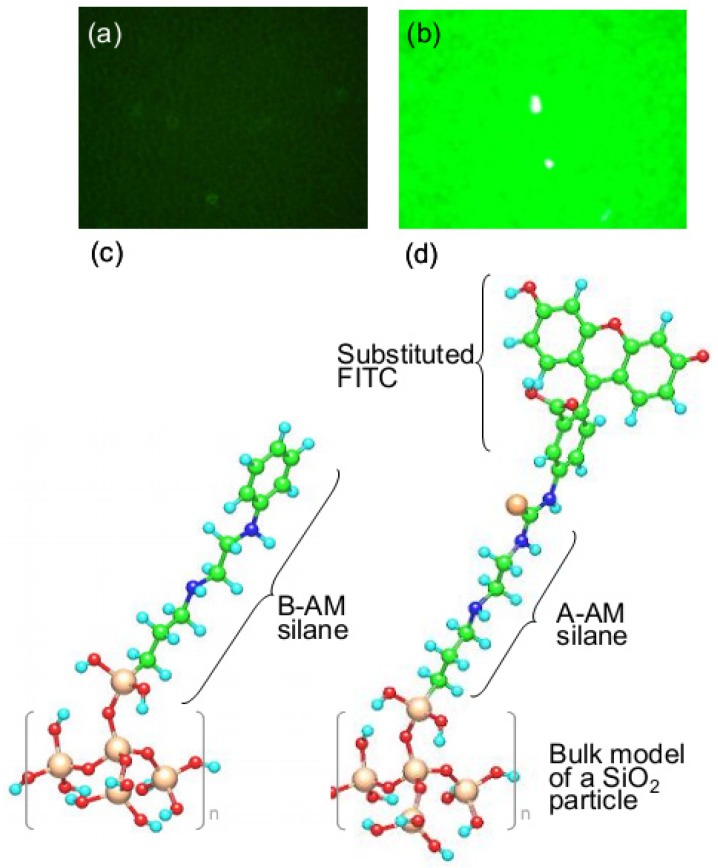
Fluorescence microscope images of (**a**) [B-AM silane (2%)]/SiO_2;_ and (**b**) [A-AM silane (2%)]/SiO_2_ after reaction with FITC. Models of the surface structure are shown in (**c**) and (**d**), respectively.

Figure 8 shows fluorescence microscope images of [B-AM silane (2%)]/SiO_2_ (Figure 8a) and [A-AM silane (2%)]/SiO_2_ (Figure 8b) after reaction with FITC, and models of the surface structure of both particles are shown in Figure 8c and d, respectively. The isothiocyanate group in FITC reacts selectively with a primary amine group, such as that in A-AM silane to form a thiourea bond between both functional groups.

The intensity of green fluorescence at λ = 520 nm from the samples is clearly different. [A-AM silane (2%)]/SiO_2_ treated with FITC shows a relative fluorescence intensity of 250 ± 4, whereas for [B-AM silane (2%)]/SiO_2_ it is 50 ± 4. These results suggest that the terminally-connected phenyl group in B-AM silane and amino group in A-AM silane are oriented outwards on the surface of the SiO_2_ particles. 

### 3.3. Evaluation for Interaction of Single-stranded Nucleic Acids and Fluorescence Compound

**Figure 9:**
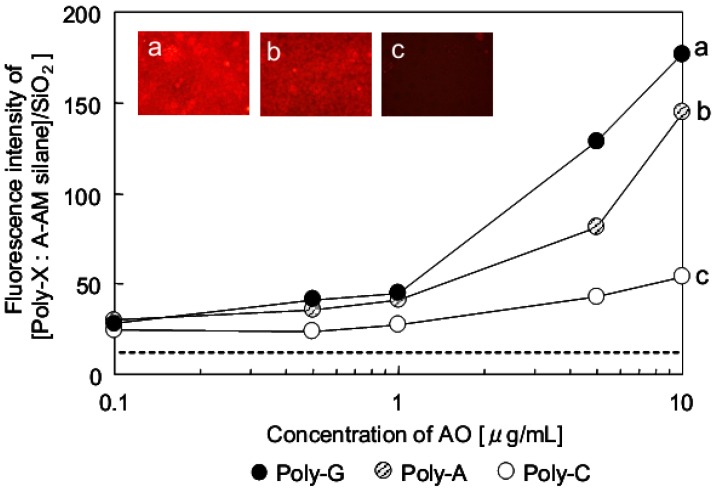
Relationship between concentration of AO and fluorescence intensity of [AO:Poly-X:A-AM silane]/SiO_2_ [Poly-X: (**a**) X = guanine; (**b**) adenine; (**c**) cytosine].

Figure 9 shows the relationship between the concentration of AO and fluorescence intensity of Poly-X (X = guanine (G), adenine (A) and cytosine (C)) conjugated with [A-AM silane]/SiO_2_. The schematic illustrations are also shown in Figure 4c. The fluorescence intensity of [A-AM silane]/SiO_2_ treated with AO was measured to confirm their interaction. There was no interaction between AO and [A-AM silane]/SiO_2_ (dotted line in Figure 9). The intensity of the red fluorescence at λ = 640 nm clearly depended on the type of polynucleotide in the conjugate. When different concentrations of AO were conjugated with [Poly-G:A-AM silane]/SiO_2_, the relative fluorescence intensity of [AO:Poly-G:A-AM silane]/SiO_2_ was 45 for 1 μg/mL, 129 for 5 μg/mL and 177 for 10 μg/mL. When AO was conjugated with [Poly-A: A-AM silane]/SiO_2_, the fluorescence intensity of [AO: Poly-A: A-AM silane]/SiO_2_ decreased compared with that of [AO: Poly-G: A-AM silane]/SiO_2_ to 41 for 1 μg/mL, 81 for 5 μg/mL and 144 for 10 μg/mL. In the case of Poly-C, the fluorescence intensity of [AO:Poly-C:A-AM silane]/SiO_2_ increased very little with concentration, showing an intensity of 27 for 1 μg/mL, 43 for 5 μg/mL and 54 for 10 μg/mL. 

The sensitivities of fluorescence intensity, *R*, of AO-labeled [Poly-X: A-AM silane]/SiO_2_ conjugates are summarized in Table 1. For comparison, *R* of AO-labeled Poly-X ([AO: Poly-X]) are also summarized in Table 1. *R* was calculated using Equation (1):

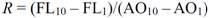
(1)

**Table 1:** Sensitivities for fluorescence intensity of AO-labeled (**a**) [Poly-X:A-AM silane]/SiO_2_ particles and (**b**) polynucleotides.

single-stranded nucleic acid	[AO:Poly-X:A-AM silane]/SiO_2_			[AO:Poly-X]		
	FL_10_	FL_1_	R^(1)^	FL_10_	FL_1_	R^(1)^
Poly-G	177	45	14.7	141	37	11.6
Poly-A	144	41	11.4	130	20	12.2
Poly-C	54	27	3.0	40	27	1.4

Note: (1) *R* = (FL_10_ – FL_1_)/(AO_10_ − AO_1_).

In this instance, AO_10_ and AO_1_ were the concentrations of AO (μg/mL), while FL_10_ and FL_1_ were the fluorescence intensities when the concentrations of AO were 10 and 1 μg/mL, respectively. The difference of conjugation ability is particularly clear when single-stranded nucleic acids trapped with aminoalkylated SiO_2_ are used. *R* of [Poly-X:A-AM silane]/SiO_2_ increased in the order Poly-C < Poly-A < Poly-G. A similar trend was obtained for the [AO:Poly-X] complexes (amino-alkylated SiO_2 _particle free). These results suggest that the conjugation of AO and [Poly-X:A-AM silane]/SiO_2_ is affected by the characteristics of the single-stranded nucleic acid. Based on the above results, it estimates that the difference of fluorescence intensity for AO and [Poly-X:A-AM silane]/SiO_2_ was obtained clearly when the amount of each poly-X on amino-alkylated SiO_2_ particle was the same.

### 3.4. Evaluation of the Interaction Between Nucleic-Acid Base in Nucleotides and Fluorescent Compound

Electron distributions of each model structure, which a surface model of a [A-AM silane]/SiO_2_ cluster (Figure 6b), Poly-G, A or C with 3 units as the nucleotide fragment (Table 2) and fluorescence compound (Figure 4b), were calculated separately by the PM3 method. 

Based on the results of electron distributions, the highest occupied molecular orbital (HOMO) is distributed on the terminal of each [A-AM silane]/SiO_2_ cluster. This is because the nitrogen atom in an amino group has a lone electron pair. This result suggests that the surface of aminoalkylated SiO_2_ has an electron-donating ability. In PBS solution (pH = 7), protonation of the primary amino group is an unfavorable reaction. It estimates there is little positive electric charge on the surface of the silica particle.

In contrast, it has been reported that AO as a fluorescence compound interacts with the phosphate group on the nucleotide by electrostatic interaction [13,14]. In accordance with the MO calculation result, the HOMO of AO, which contains a π electron, is also delocalized over the entire molecule. This suggests that AO also possesses electron-donating ability. It estimates that AO attaches to a phosphate group in the nucleotide by electrostatic interaction first, then the nucleobase around the AO interacts through its electron-donating ability.

Based on the results in Figure 9 and Table 1, the polynucleotides conjugate was trapped with aminoalkylated SiO_2_ and was further conjugated with AO. This suggests that the polynucleotide behaves as an electron acceptor, and two vacant orbitals are necessary to interact with both aminoalkylated SiO_2_ and AO. Based on the results of electron distributions, the lowest unoccupied molecular orbital (LUMO) and second LUMO of the nucleotide fragment are distributed on a central nucleotide and 5’ end.

The interaction between the nucleotide and AO was evaluated using Equation (2):

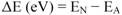
(2)

In this instance, E_N_ is the energy level of the LUMO obtained from a MO calculation of each nucleotide, E_A_ is energy level of the HOMO obtained from the MO calculation of AO, and ΔE is an energy gap between each nucleotide conjugated and AO. Therefore, it is possible to determine an interaction parameter between two molecules using Frontier orbital theory [16].

A comparison of ΔE values is given in Table 2. ΔE decreased in the order of Poly-C > Poly-A > Poly-G, consistent with the results presented in Figure 9. This order reflects the interaction between fluorescent compounds and the nucleobase in single-stranded nucleic acids.

**Table 2:** Calculated energy levels for Poly-X^(2–4) ^and AO.

Calculated MO	AO^(1)^	Poly-C^(2)^	Poly-A^(3)^	Poly-G^(4)^
HOMO [eV]	−7.695	−9.140	−8.840	−8.820
LUMO [eV]	−0.819	−0.345	−0.449	−0.482
ΔE	–	7.350	7.246	7.213
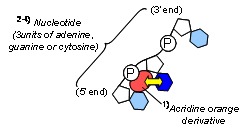				

## 4. Discussion

In general, an adsorbent adsorbs a molecule through molecular interactions such as Van der Waals’ forces. As a result, there is no selectivity for adsorption of molecules. However, aminoalkylated SiO_2_ is trapped with a single-strand nucleic acid that is further conjugated to AO through hydrogen bonding and charge-transfer interactions, respectively. Target molecules are immobilized arbitrarily on the surface of aminoalkylated SiO_2_ by hydrogen bonding. Levit-Binnun *et al*. used a quantitative approach to explain the interaction between immobilized BSA on a solid surface and a biotinylated anti-BSA antibody as a target molecule [17]. They immobilized BSA on a substrate and then reacted it with a biotinylated anti-BSA antibody. The amounts of bonding m_D_ were evaluated by Equation (3):

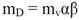
(3)

where α is the formation efficiency of a complex between immobilized protein P and target molecule M, β is the detection efficiency, and m_v_ is the number of M that can interact with P.

When M was labeled with a fluorescent compound, β became 1, allowing Equation (3) to be simplified to:

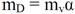
(4)
where α is an eigenvalue that mainly depends on the surface coverage of functional group σ, the immobilization rate of P on the surface ρ and the affinity between P and M, K_d_.

In this study, P and M correspond to Poly-X and AO, respectively, and m_D_ was approximated using Equation (4). M was constant, therefore, it is estimated that m_v_ was also constant. σ was 100% because the SiO_2_ surface saturated with A-AM silane during coupling. Experimental results showed that ρ did not depend on the type of polynucleotide, so was a constant. As a result, α did not depend on ρ. 

In contrast, K_d_ was significantly influenced by the type of polynucleotide, which is consistent with both experimental and simulation results. This suggests that the surface properties of aminoalkylated SiO_2_ strongly reflect chemical properties such as the electron density on molecule P that can conjugate to the surface.

## 5. Conclusions

In this study, single-stranded nucleic acids were trapped on a solid surface using hydrogen bonding, which prevented denaturation of the biopolymers. The surface of silica particles was modified with A-AM silane to form primary amines that interact with single-stranded nucleic acids through hydrogen bonding to form conjugated materials in a short time. This complex was then selectively interacted with a fluorescence labeling reagent such as an acridine orange derivative. Changes in fluorescence intensity depended on the polynucleotide with the order poly-guanine > poly-adenine >> poly-cytosine. This suggests the different fluorescence intensities reflected the different molecular interactions between the acridine orange derivative and nucleic-acid base in polynucleotides. The results of MO calculations suggest that the surface properties of aminoalkylated SiO_2_ strongly reflect its chemical properties, such as the electron density of a molecule that can conjugate to the surface. 

## Abbreviations

A-AM silane: N-2-aminoethyl-3-aminopropyltrimethoxysilane (KBM-603, Shin-Etsu Chemical Co., Ltd., Tokyo, Japan)
B-AM silane: N-phenyl-3-aminopropyltrimethoxysilane (KBM-573, Shin-Etsu Chemical Co., Ltd., Tokyo, Japan)
[A-AM silane]/SiO_2_: silica particles surface-modified with aminoalkyl chains
[X-AM silane]/SiO_2_: X = A: amino group and B: phenyl group
Poly-X: polynucleotides-X, X = A (adenine), G (guanine) and C (cytosine)
AO: derivatives of acridine orange (Cellstain-AO; Dojindo Laboratories, Kumamoto, Japan).)
FITC: fluorescein isothiocyanate
MO: Molecular orbital
HOMO: highest occupied molecular orbital
LUMO: lowest unoccupied molecular orbital
